# Rapid onset avascular necrosis secondary to misuse of appetite stimulant

**DOI:** 10.1186/s40337-019-0257-z

**Published:** 2019-08-01

**Authors:** Hilary Stevenson, Salome Cockern, Elizabeth Secord

**Affiliations:** Wayne State University School of Medicine, Horizons Project, 4201 St Antoine, Detroit, MI 48201 USA

**Keywords:** HIV, Antiretroviral, Avascular necrosis, Megestrol, Appetite stimulants

## Abstract

**Background:**

HIV patients often fear disclosure of their status and consequently are fearful that their appearance may lead people to believe they have HIV. Excessive attention to weight is one result.

**Case presentation:**

We present a case of bilateral avascular necrosis (AVN) of the femoral heads secondary to megestrol misuse. Multiple providers with non-communicating electronic medical records contributed to this event, especially in our patient who was a guarded historian. Because of altered body image and fear of HIV disclosure, our patient who was already prescribed an antiretroviral associated with decreased bone density, sought an appetite stimulant and used far more than the prescribed dose leading to rapid onset AVN.

**Conclusions:**

Use of appetite stimulants in HIV should be carefully monitored and utilized only when weight loss is excessive and documented, and steroid based appetite stimulants should be avoided.

## Lay summary

Body dymorphic disorder can result in some cases in an obsessive feeling of being too thin. Our patient who is HIV+ did not have all criteria needed to diagnose body dysmorphic syndrome, but excessive anxiety led him to be concerned that people would know his status because of thinness. This resulted in overuse of appetite stimulants with side effects on his bones, causing damage that would eventually require hip replacement. Our inability to see other providers’ prescriptions prevented our surveillance for this problem.

## Case report

An 18 year old was diagnosed with human immunodeficiency virus (HIV) by routine testing at his pediatrician’s office and was referred to adolescent HIV clinic. His initial concern at diagnosis was some diarrhea that resolved without intervention. He remained worried about the weight loss from the diarrhea because he had always been what he considered to be “too thin”. His weight returned to baseline by his second visit to our clinic. He reported prior diagnoses of schizophrenia and bipolar disorder in childhood, and was taking olanzapine and aripiprazole. He denied use of tobacco, alcohol or any recreational drugs. He delayed starting antiretroviral therapy for 2 years due to fear of side effects. He had read about atazanavir on the internet, and knew that discoloration of the eyes was a side effect. We discussed other options but he remained very fearful. He was aware of his viral load (26,400 copies/ml) and his CD4+ T cell count (660 cells/ul), and deferred antiretroviral therapy (ART).

At the age of 20 years, after some therapy sessions related to anxiety, he agreed to begin ART, initiating with emtricitabine-tenofovir, darunavir and ritonavir. His CD4+ T cell count at that time was 432 cells/ul (16%) and his HIV viral load count was 14,022 copies/ml. Four months later his CD4+ T cell count was 884 cells/ul and his HIV viral load was undetectable.

At the age of 23 the patient began requesting an appetite stimulant, stating that he was still “too thin”. His height was 172 cm, weight 59Kg and body mass index (BMI) was 19 (normal 18.5–24). This data was discussed with him and we did not prescribe an appetite stimulant, but gave dietary recommendations for increasing weight and referred to a dietician. Two months later he began reporting knee and ankle pain from “exercise”. He began to develop various joint complains including heel, knee and right hip pain. The right hip pain persisted for several months. He subsequently began to appear cushinoid and his BMI increased to 23. He had developed moon facies and striae on his abdomen.

At this time he reported obtaining a prescription 4 months earlier for 40 mg a day of megestrol from his primary care doctor to “improve my appetite because I have HIV”. He further reported that he had increased the dose to 400 mg a day to make it work faster because he was fearful that people would see him looking thin and think he had acquired immunodeficiency syndrome (AIDS).

**Fig. 1:**
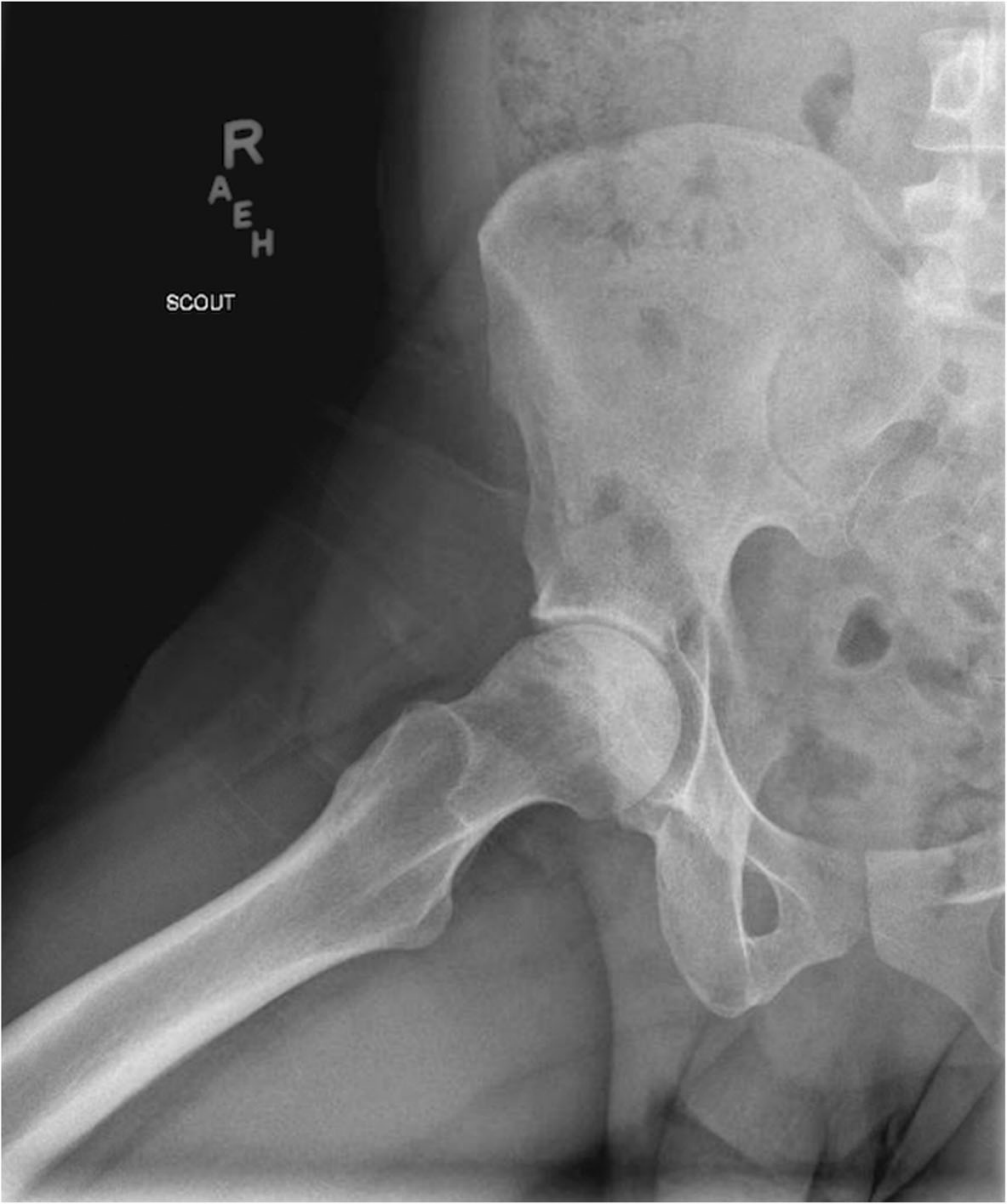
Right hip arthrography reveals femoral head osteonecrosis without subchondral collapse

An x-ray of his hips with arthroscopy confirmed bilateral femoral head osteonecrosis with increased degeneration on the right side (Fig. 1). Surgery was recommended but he was fearful of surgery and would not consider it at that time.

## Discussion

Our patient developed signs and symptoms of bilateral AVN of the hip after just 4 months of misusing the synthetic progestin appetite stimulant megestrol combined with ART containing tenofavir disoproxil. This example illustrates the need to be cautious in prescribing and monitoring of steroid based appetite stimulants in any patient and suggests a need for stringent criteria in selecting patients who will benefit from any appetite stimulants [1–4].

Central nervous system drugs, growth factors, and medical marijuana, or marijuana derivatives such as dronabinol are non-steroidal appetite stimulants [5, 6]. These agents may be more appropriate alternatives for cases of life threatening weight loss. The most appropriate intervention in cases where life is not at risk from severe weight loss is dietary change.

Higher than normal instances of osteonecrosis has been reported in the HIV population [7]. One risk factor for HIV patients is antiretroviral therapy with the older form of tenofavir, i.e. tenofavir disoproxil, which was associated with numerous reports of osteonecrosis of multiple joints [7]. Our patient was also on tenofavir disoproxil.

This case illustrates the need to be judicious with use of appetite stimulants in HIV patients, and complete avoidance of steroid based appetite stimulants. In the age of good ART, the need for appetite stimulants is rare, although the request for them remains high. Education about healthy weight, diet, and medication complications is key.

## Data Availability

Not applicable-no data sets were generated or analyzed for this case report.

## Abbreviations

AIDS: acquired immunodeficiency syndrome
ART: antiretroviral therapy
AVN: avascular necrosis
BMI: Body Mass Index
HIV: Human Immunodeficiency Virus
